# Estimating the true effectiveness of smoking cessation interventions under variable comparator conditions: A systematic review and meta‐regression

**DOI:** 10.1111/add.16222

**Published:** 2023-05-24

**Authors:** Jannis Kraiss, Wolfgang Viechtbauer, Nicola Black, Marie Johnston, Jamie Hartmann‐Boyce, Maarten Eisma, Neza Javornik, Alessio Bricca, Susan Michie, Robert West, Marijn de Bruin

**Affiliations:** ^1^ Radboud Institute for Health Sciences Radboud University Medical Centre Nijmegen The Netherlands; ^2^ Department of Psychology, Health, and Technology University of Twente Enschede The Netherlands; ^3^ Department of Psychiatry and Neuropsychology Maastricht University Maastricht The Netherlands; ^4^ Institute of Applied Health Sciences, Health Psychology Group University of Aberdeen Aberdeen UK; ^5^ Nuffield Department of Primary Care Health Sciences University of Oxford Oxford UK; ^6^ Department of Clinical Psychology and Experimental Psychopathology University of Groningen Groningen The Netherlands; ^7^ Department of Sports Science and Clinical Biomechanics, Research Unit for Musculoskeletal Function and Physiotherapy University of Southern Denmark Odense Denmark; ^8^ Department of Physiotherapy and Occupational Therapy, The Research Unit PROgrez Næstved‐Slagelse‐Ringsted Hospitals Slagelse Denmark; ^9^ Centre for Behaviour Change University College London London UK; ^10^ Department of Epidemiology and Public Health, Health Behaviour Research Centre University College London London UK

**Keywords:** behavioural interventions, comparators, effectiveness, meta‐regression, randomised controlled trial, smoking cessation

## Abstract

**Background and aims:**

Behavioural smoking cessation trials have used comparators that vary considerably between trials. Although some previous meta‐analyses made attempts to account for variability in comparators, these relied on subsets of trials and incomplete data on comparators. This study aimed to estimate the relative effectiveness of (individual) smoking cessation interventions while accounting for variability in comparators using comprehensive data on experimental and comparator interventions.

**Methods:**

A systematic review and meta‐regression was conducted including 172 randomised controlled trials with at least 6 months follow‐up and biochemically verified smoking cessation. Authors were contacted to obtain unpublished information. This information was coded in terms of active content and attributes of the study population and methods. Meta‐regression was used to create a model predicting smoking cessation outcomes. This model was used to re‐estimate intervention effects, as if all interventions have been evaluated against the same comparators. Outcome measures included log odds of smoking cessation for the meta‐regression models and smoking cessation differences and ratios to compare relative effectiveness.

**Results:**

The meta‐regression model predicted smoking cessation rates well (pseudo R^2^ = 0.44). Standardising the comparator had substantial impact on conclusions regarding the (relative) effectiveness of trials and types of intervention. Compared with a ‘no support comparator’, self‐help was 1.33 times (95% CI = 1.16–1.49), brief physician advice 1.61 times (95% CI = 1.31–1.90), nurse individual counselling 1.76 times (95% CI = 1.62–1.90), psychologist individual counselling 2.04 times (95% CI = 1.95–2.15) and group psychologist interventions 2.06 times (95% CI = 1.92–2.20) more effective. Notably, more elaborate experimental interventions (e.g. psychologist counselling) were typically compared with more elaborate comparators, masking their effectiveness.

**Conclusions:**

Comparator variability and underreporting of comparators obscures the interpretation, comparison and generalisability of behavioural smoking cessation trials. Comparator variability should, therefore, be taken into account when interpreting and synthesising evidence from trials. Otherwise, policymakers, practitioners and researchers may draw incorrect conclusions about the (cost) effectiveness of smoking cessation interventions and their constituent components.

## INTRODUCTION

In trials evaluating the effectiveness of behavioural interventions, such as smoking cessation or type 2 diabetes self‐management, interventions are typically evaluated against an active comparator [1, 2, 3, 4]. Preliminary research suggests that comparators (or controls, we use the term comparator) can vary substantially between trials and affect intervention effect sizes [5, 6, 7]. This implies that comparator variability would need to be taken into account when interpreting, synthesising or generalising intervention effects reported in trials. This is, however, not common practice and complicated by the poor reporting of comparators in the reports of trials [5]. The current systematic review project builds on an early, small‐scale meta‐analysis exploring these issues [6] and was designed to provide more definitive evidence on the importance of accounting for comparator group variability in behavioural intervention trials. This could have implications for the reporting and synthesis of behavioural intervention trials and on conclusions from systematic reviews and economic models regarding which behavioural interventions should be considered for implementation in routine health services. The current meta‐analysis uses a large database on smoking cessation trials to examine this issue. Hence, in addition to methodological evidence, this study should provide more accurate evidence regarding the effectiveness of behavioural smoking cessation interventions.

### Smoking cessation intervention trials

Tobacco smoking is a leading cause for premature mortality and disease and is associated with substantial healthcare costs [8, 9]. Several previous meta‐analyses have synthesised data on the effectiveness of different types of behavioural smoking cessation interventions [10, 11, 12, 13, 14, 15, 16, 17, 18, 19]. Usually, these meta‐analyses compare effect sizes such as (standardised) mean differences or odds ratios, contrasting the outcomes from experimental and comparator (control) groups, to draw conclusions about whether interventions work and, which type of interventions seem to work best. In behavioural intervention trials, however, experimental interventions are typically evaluated against an active comparator, often described in scientific articles as receiving ‘usual care’, ‘brief advice’ or ‘education only’. In a small meta‐analysis of behavioural interventions in HIV care, we have previously found that comparators—all described as receiving care‐as‐usual—were exposed to behavioural support that varied widely between trials, affected comparator outcomes and therefore, trial effect sizes [6]. Accounting for these comparator differences resulted in fairer comparisons of the effectiveness of these interventions and revealed an overall underestimation of intervention effectiveness compared with ‘traditional’ bivariate meta‐analyses.

Given that comparator variability is often ignored in systematic reviews and meta‐analyses of behavioural interventions, we decided to advance this early work in a large confirmatory systematic review and meta‐analysis of behavioural smoking cessation trials, which we present here.

### The active content of smoking cessation interventions and comparators

In smoking cessation trials, interventions can be roughly divided into three components: (1) smoking cessation medication; (2) smoking cessation behavioural support, including smoking cessation information and motivational/behaviour planning techniques; and (3) adjuvant interventions that focus on smoking cessation indirectly, for example, through relaxation techniques or weight management. These interventions can be delivered in‐person (individually, group) or in writing (e.g. leaflets, websites), by different providers (e.g. counsellors, nurses and physicians), and vary in terms of content (i.e. the behaviour change techniques [BCTs] used [such as goal setting or self‐monitoring], individually tailored or not, and the behaviours targeted) and exposure times (i.e. the number and duration of smoking cessation sessions). Smoking cessation interventions—experimental and comparators—may therefore, vary from very brief, simple and cheap (such as an information leaflet) to intensive, sophisticated and resource‐intensive group interventions delivered by highly qualified staff over an extensive time period.

In a previous meta‐analysis of 141 smoking cessation trials (a subset of the studies included in the current meta‐analysis), we examined whether variability in smoking cessation support provided to comparator groups, predicted comparator group cessation rates [1]. We indeed found that both medication provision and number of BCTs delivered to comparator group participants predicted smoking cessation and explained up to 15% point differences in cessation rates between comparator groups (range, 8%–23%). There is, therefore, good reason to suspect that accounting for this variability in comparators should lead to more valid comparisons of the effectiveness of smoking cessation interventions across trials.

### Reporting of smoking cessation interventions

To examine whether comparators affect trial effect sizes, it is important to have sufficient information on the interventions provided in all trial arms. Despite CONSORT requiring the full reporting of interventions [20], underreporting of interventions is still very common [5]. In preparatory research, we have, therefore, gone through the time consuming process of, first, identifying all of the already‐published trial materials and then (over a period of more than a year) contacting all trial authors to retrieve additional unpublished materials on both the experimental and comparator interventions. In these unpublished materials, we identified, for example, an additional 70% of smoking cessation BCTs that were not included in any of the published trial materials [5]. Descriptions of comparator interventions in published materials were poorer than those for experimental interventions, and despite improved intervention reporting guidelines becoming increasingly available [7, 21], there was no evidence that intervention reporting improved with time. Nevertheless, through contacting trial authors, we were able to obtain full information for 70% of the experimental and 77% of the comparator interventions [5].

### Prior meta‐analysis of smoking cessation interventions

Many previous meta‐analyses have examined the relative effectiveness of different types of smoking cessation interventions [10, 11, 12, 13, 14, 15, 16, 17, 18]. Whereas most reviews ignore differences in comparators, some have accounted for differences in comparators to some extent using two methods. First are pairwise meta‐analyses in which effect sizes from trials that used different comparators (e.g. no‐intervention controls, self‐help, brief support or intensive individual counselling) to evaluate the effectiveness of one type of intervention (e.g. group interventions) were synthesised separately. These generally reveal that intervention effects become smaller as the comparator was more intensive. Limitations of these meta‐analyses are that they cluster comparators into broad categories (e.g. self‐help, intensive counselling) that can still include substantial variability in active content (i.e. provision of medication and BCTs) [1, 6]. Moreover, they cannot compare all possible combinations of experimental and comparator conditions. For example, e‐health intervention trials rarely have intensive individual counselling as comparator, but many trials evaluating experimental group interventions have individual counselling as the comparator. Such meta‐analyses are also left with a limited number of studies when analysing specific combinations of experimental interventions and comparators, decreasing power and precision.

The second method is a network meta‐analysis, including one of behavioural smoking cessation interventions, which was recently published by a co‐author [19]. Although highly informative, this study examined which of 38 individual intervention components were associated with increased smoking cessation (when compared with ‘minimal intervention’), but did not look at the impact of differences in comparators between smoking cessation trials on conclusions about their (relative) effectiveness. A relevant limitation of this network meta‐analysis is that it relied on published information about interventions only. Yet, recent studies have found that on average approximately 70% of the information on the active content of behavioural smoking cessation interventions (i.e. BCTs) is not reported, yet predicts smoking cessation outcomes [1, 5]. Taking into account these unpublished data about experimental and comparator interventions is, therefore, likely to provide more valid and precise estimates of the effectiveness of different types of behavioural interventions and their constituent components.

### Research aims

Decision makers weigh the costs and benefits of different interventions to make their decision on which interventions to fund and implement. It is, therefore, important that systematic reviews and meta‐analyses make a fair comparison of the effectiveness of interventions categories (e.g. are individually delivered interventions more effective than group‐delivered interventions?) and of interventions within a given category (e.g. which of the group interventions are most effective?). These comparisons may however be obscured because comparators may vary between trials and intervention categories and because experimental and comparator interventions are poorly reported, making it difficult to estimate and compare the true effectiveness of different types of interventions.

The objectives of this study were to examine the impact of variability in comparators on conclusions about the effectiveness of different behavioural smoking cessation interventions, at the level of individual intervention trials and at the level of intervention categories using comprehensive data on the experimental and comparator interventions. First, we aimed to build a meta‐regression model that is both parsimonious and predicts trial smoking cessation rates well. Second, we used this model to re‐estimate trial effect sizes, as if all smoking cessation interventions had been evaluated against the same comparators. All objectives, hypotheses, analyses and statistical models were pre‐registered (https://osf.io/23hfv/). For completeness, these steps and hypotheses are explained in the analyses section of this manuscript.

## METHODS

This study is part of the IC‐SMOKE project, a large systematic review of smoking cessation trial methodology [22]. The project is also registered on the International Prospective Register of Systematic Reviews (PROSPERO) (CRD42015025251) and the Open Science Framework (OSF) (https://osf.io/23hfv/). A completed Preferred Reporting Items for Systematic Reviews and Meta‐analyses (PRISMA) checklist [23] can be found in Appendix S1.

### Eligibility

The Cochrane Tobacco Addiction Group Specialised Register was searched (first in November 2015, update in October 2018) for RCTs assessing the impact of behavioural interventions (with or without pharmacological support) on biochemically verified smoking cessation at 6 months or longer. Inclusion criteria were published after 1996 (contacting authors for trials published >20 years ago was judged to be unrealistic), in English, in peer‐reviewed journals and targeting adult smokers (18 years or older). Although the final search has been conducted some years ago, given the methodological focus of the current study and the time‐consuming data collection and coding methods, an updated literature search was not deemed feasible or necessary.

### Procedure

Intervention data were first extracted from published materials, including primary articles, appendices, protocols, intervention development papers and trial websites. Next, we initiated the process of contacting the authors of the articles to retrieve unpublished intervention materials [5]. This was first done via email (including two reminders) to the first author, then to the second and last authors and finally middle authors, as required, including a reminder.

Authors were asked to send any additional intervention materials regarding experimental and comparator groups, such as manuals, practitioner training materials, self‐help materials or website content. For comparator interventions (e.g. usual care), authors often lack materials describing the smoking cessation support provided. To address this issue, we developed a dedicated comparator intervention checklist (https://osf.io/e834t/), which authors were asked to complete online. We had previously shown that this method produced reliable and valid data in another domain [6, 24], as well as in our previous study on smoking cessation comparator groups [1].

### Data extraction

Two trained researchers independently and reliably [25] extracted intervention characteristics from all available materials. This included using the BCT taxonomy (BCTTv1) (Table SA2) [26, 27] for coding the presence or absence of 93 different BCTs. A sum score of the number of BCTs targeting (1) quitting smoking and (2) abstaining from smoking was calculated, serving as the ‘active content score’. Hence, BCTs that were delivered both for quitting and abstaining were counted twice. We also coded whether a BCT was personalised to individual clients, whether pharmacological support or adjuvant interventions were delivered, exposure time, the mode of delivery, number of sessions and if the respective group was an experimental or comparator group (see Table 1). Coders also independently and reliably assessed whether interventions were sufficiently well described to assume full information was available on all intervention components.

**TABLE 1:** Description and coding of all variables included in meta‐regression models.

**Active elements**		
Total BCTs	Total number of quitting and abstinence BCTs provided to the group. Sum of personalised and non‐personalised BCTs	Continuous
Personalised BCTs	Number of quitting and abstinence BCTs that were individually tailored or required active involvement by the participant	Continuous
Non‐personalised BCTs	Number of quitting and abstinence BCTs that were not individually tailored or did not require active involvement by the participant	Continuous
Pharmacological support	Whether the sample received medication.	Categorical (0 = no, 1 = yes)
Adjuvant interventions	Whether an intervention was provided that was hypothesised by the study authors to either indirectly lead to smoking cessation or have benefits as an add‐on to an existing smoking cessation programme and that was not captured by any of the other active content predictors	Categorical (0 = no, 1 = yes)
Log‐transformed exposure time	Number of minutes that participants received an intervention	Continuous
Mode of delivery	Whether the interventions were delivered interpersonally or in writing^a^	Categorical (0 = written, 1 = person‐delivered)^a^
Group type	Whether the respective group was a comparator or experimental group within the trial	Categorical (0 = comparator, 1 = experimental)
**Pre‐defined covariates**		
Age	Mean age of sample	Continuous
Nicotine dependence	Mean Fagerström Test for Nicotine Dependence of the group. Missing values were imputed based on cigarettes per day where available	Continuous
Log‐transformed length of follow‐up	Number of half years post‐randomisation	Continuous
Cotinine verification of smoking cessation	Whether cotinine verification of abstinence was used	Categorical (0 = no, 1 = yes)
Type of abstinence assessed	Whether abstinence was assessed as sustained or point prevalence	Categorical (0 = prevalence, 1 = sustained)
**Optional covariates**		
Physical condition	Whether participants in the group were (mainly) recruited with chronic health issues such as heart or respiratory conditions	Categorical (0 = no, 1 = yes)
Mental health challenges	Whether the majority of participants of the group had mental health problems (e.g. substance use disorders, depression)	Categorical (0 = no, 1 = yes)
Physical health trigger	Whether participants were recruited with a health‐related trigger for smoking cessation	Categorical (0 = no, 1 = yes)
Attrition	The proportion of participants lost from the sample at follow‐up^b^	Continuous

Abbreviation: BCTs, behaviour change techniques.

^a^ Mode of delivery was coded differently for the subgroup analyses: interpersonal subgroup analysis (0 = individual, 1 = group), written subgroup analysis (0 = paper‐based, 1 = digital).

^b^ Attrition proportion was calculated as follows: The sum of number of participants who were not able to be found for follow‐up, the number of participants who miscarried (in studies of smoking cessation during pregnancy) and the number of participants who otherwise did not provide abstinence data, divided by the total number of participants within that group.

Data on multiple pre‐defined covariates were also extracted. These included (1) mean age; (2) mean nicotine dependence of the group; (3) length of follow‐up; (4) cotinine verification of abstinence (yes/no); and (5) whether the outcome reflected point prevalence or sustained smoking cessation (0 = prevalence, 1 = sustained). Smoking cessation rates are typically reported assuming that missing cases are smokers, which is the standard in this field and also used by the Cochrane Tobacco Addiction Group [28]. However, as this assumption may not be accurate and introduce bias in the results (e.g. treatment groups with higher dropout will have lower smoking cessation rates), we also extracted attrition data to enable controlling for this in the analyses. Data were also extracted about physical condition, mental health challenges and physical health triggers to control for in the sensitivity analyses (for a complete overview see Table 1). Our analysis plan includes the rationale for each of these variables and the empirical support behind including them in our models (https://osf.io/khm8u/).

### Data analyses

In line with the pre‐published analysis plan (https://osf.io/khm8u/), only experimental and comparator interventions that were coded as ‘Well‐described’ were included in the analyses. We used all timepoints from 6‐month post‐randomisation and onward in bivariate mixed‐effects meta‐regression models, using the metafor package in R [29, 30]. Random effects were included for trials and groups to account for clustering of groups within trials and timepoints within groups to account for dependency in repeated assessments within the same group. For the latter, we assumed that the random effects are autocorrelated using a continuous‐time autocorrelation (CAR) structure. The dependent variable in all models was the logit‐transformed smoking cessation rates in the respective treatment arm. For the sampling errors of these rates, we also assumed a CAR structure, with a conservative estimate 0.9 for the autocorrelation of two rates 1 month apart. The following a priori defined control variables were included simultaneously in each model: (1) mean age of the respective treatment group; (2) mean nicotine dependence score of the respective treatment group; (3) log‐transformed length of follow‐up of the study; (4) carbon monoxide (CO) versus cotinine verification in the study; and (5) type of abstinence assessed (point prevalence or sustained abstinence) in the study. R scripts, the dataset and outputs are available on the OSF website (https://osf.io/23hfv/). All models were checked for outliers and influential cases using standardised residuals, leverages and Cook’s distance.

#### Step 1: building the model

**FIGURE 1:**
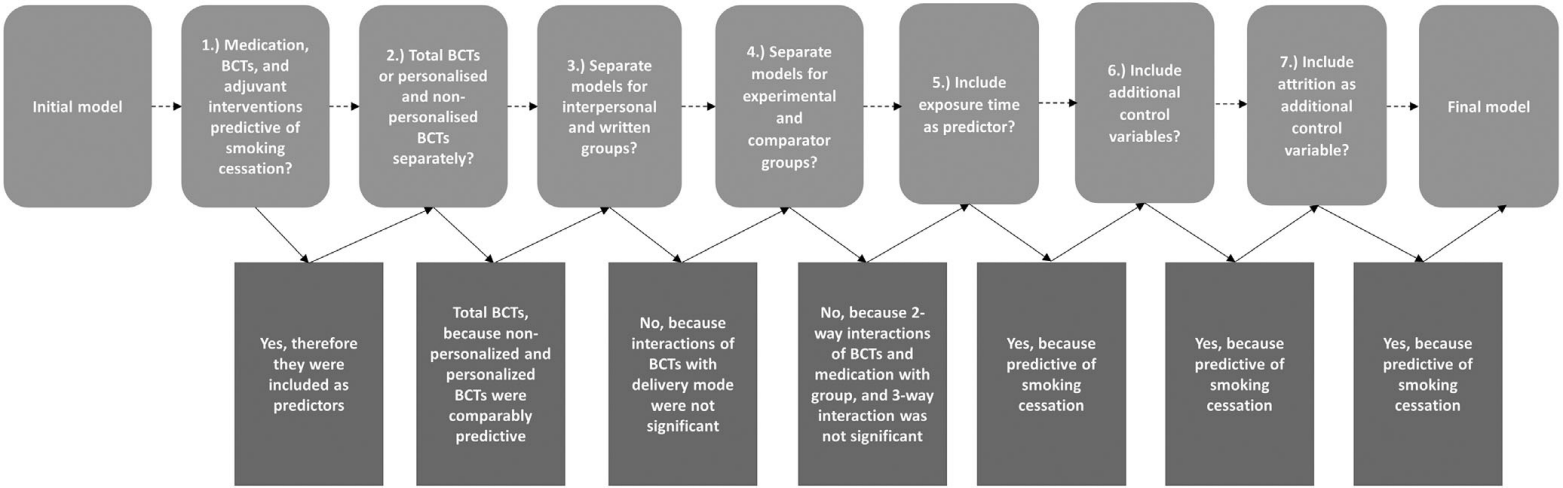
Decision flowchart summarising the decisions taken in the course of the analyses leading to the final model used for the re‐estimation of smoking cessation rates. The top row reflects the order of decisions made. The bottom row reflects the outcomes of this decision process leading to the final model.

The first step in the analysis was to build a model that accurately predicts cessation rates in experimental and comparator groups. This was done by fitting a series of multiple bivariate mixed‐effects meta‐regression models, based on pre‐defined hypotheses and decision criteria (see top row of Figure 1). Following Figure 1 from left to right, we first examined (step 1 of model 1, Table SB1) whether the provision of smoking cessation medication (pharmacological support), a BCT total score (the sum score of smoking cessation BCTs for quitting and abstinence) and adjuvant interventions provided to experimental or comparator groups predicted smoking cessation rates in the respective trial arms. Second, we examined whether total BCTs or personalised and non‐personalised BCTs separately should be included in the model (step 1 of model 2, Table SB1). Third, interaction terms were included to examine the hypothesis that BCTs delivered in‐person would be more effective than those delivered in writing (step 2 of model 1, Table SB1). Fourth, it was examined whether effects of medication and BCTs differed between trial arms (step 3 of model 1, Table SB1) by examining the interaction of BCTs and medication with group and by including a three‐way interaction of BCTs, delivery mode and group to examine whether the effects of BCTs depended on mode of delivery and treatment arm (step 4 of model 1, Table SB1). Fifth, a model was run with interpersonal interventions only and exposure time included to see if exposure time predicts smoking cessation (step 2 of model 1, Table SB2). The reason for running the models with interpersonal interventions separately is that it allowed for including ‘intervention exposure time’, which varied considerably between in‐person interventions, but is not available for written interventions. Sixth, sensitivity analyses were conducted with additional pre‐defined control variables (i.e. physical condition, mental health challenge and physical health trigger) (Table SB4). Seventh, a sensitivity analysis was done in which we examined if attrition predicted smoking cessation (to capture bias introduced by trials’ ‘missing is failure’ data imputation) (Table SB7). Decisions for model building were based on *P*‐values. Based on these steps, the final model was composed (for the number of studies, groups and individual comparisons, see Supporting Information).

In accordance with the analysis plan (https://osf.io/khm8u/), sensitivity analyses were also conducted with cubic splines for the BCT predictors to examine non‐linearity (for an overview of all sensitivity analyses, see Tables SB4–B9 and Figure SB1). According to the analysis plan, we also planned to repeat the analyses from the bivariate models using traditional effect sizes models, as a check. As these models yielded similar results compared with the bivariate models, they are only reported in the Supplemental materials (see Supplemental Information SB2 and Tables SB10–SB13 and Figures SB2 and SB3).

#### Step 2: re‐estimating effect sizes

The next step was to examine whether this final model predicts the observed smoking cessation rates in the included trials well. If so, the model was used for the re‐estimation of effect sizes. The procedure for re‐estimation was as follows: we pre‐defined (https://osf.io/khm8u/) seven prototypical comparator interventions and five prototypical experimental interventions, based on their mode of delivery and the person delivering the intervention (see Table 2 for the prototypes and descriptives). Similar groupings are also common in the literature, such as in Cochrane reviews [10, 11, 12, 13, 14, 15, 16, 17, 18]. We first re‐estimated effect sizes of all individual trials, as if all interventions had been trialled against the same comparators. For this, the average values for the active components (pharmacological support, number of BCTs, adjuvant interventions and exposure time) of the seven comparator groups were used to predict comparator cessation rates. The trial‐specific values were used for the experimental interventions to predict cessation rates for the experimental arms. These values were entered in the final bivariate model, resulting in seven predicted effect sizes for each trial (one for each prototypical comparator), and adjusted for the covariates in the model. Values for the covariates in the model were standardised across trials, and all effect sizes were estimated at 6‐month follow‐up using CO verification of smoking cessation. Trials’ reported effect sizes (in the trial papers) and their re‐estimated effect sizes were plotted to examine the impact of the standardising of comparators (and covariates) on conclusions about intervention effectiveness. To organise these results, trials were combined into the five prototypical experimental interventions groupings (see Table 2). This first step, therefore, allows for examining the degree to which variability in comparators affects trials’ effect sizes and affects conclusions about the effectiveness of interventions and what each (prototypical) intervention is likely to add in a variety of settings depending on the smoking cessation support currently in place. As an indication of relative effectiveness of the five types of experimental interventions versus prototypical comparators, ratio estimates for each possible combination of experimental and comparator intervention were calculated. This was done by dividing the predicted cessation rate of the experimental intervention within each trial by the predicted cessation rate of the comparator intervention. The average ratio was then calculated by taking the mean of all individual (trial‐level) ratios. In addition, 95% CI were calculated by calculating the standard error (SE) of the individual ratios and multiplying it by 1.96. This value was then used to obtain CIs for the average ratio estimate (mean ratio ± SE × 1.96).

**TABLE 2:** Clusters of prototypical experimental and comparator groups and corresponding average active content used for re‐estimation of smoking cessation rates.

Prototype	Experimental						Comparator					
	*n*	Personalised BCTs^a^	Non‐personalised BCTs^a^	Medication [n (%)]^b^	Sessions	Exposure time^c^	*n*	Personalised BCTs^a^	Non‐personalised BCTs^a^	Medication [n (%)]^b^	Sessions	Exposure time^c^
1. No support	–	–	–	–	–	–	18	0	0	0 (0)	0	0
2. Medication only	–	–	–	–	–	–	5	0	4	5 (100)	0	0
3. Self‐help	18	5	14	5 (28)	0	0	18	2	12	3 (17)	0	0
4. Physician advice	8	1	8	6 (75)	2	18	17	2	7	8 (47)	2	15
5. Nurse/pharmacist individual counselling	22	5	14	10 (46)	5	63	9	3	20	3 (33)	3	36
6. Psychologist individual counselling	59	9	14	44 (75)	8	223	37	5	19	25 (68)	6	130
7. Psychologist group counselling	33	8	16	22 (67)	11	745	10	5	22	3 (30)	8	681

Abbreviation: BCTs, behaviour change techniques.

^a^ Personalised and non‐personalised BCTs refer to the average number of personalised and non‐personalised BCTs included within each prototypical intervention.

^b^ The *n* under ‘medication’ refers to the number of groups actually receiving medication within each cluster of prototypical interventions. The percentage refers to the percentage receiving medication of all studies included in this cluster.

^c^ The unit for exposure time is minutes that people received an interpersonal intervention. For the bivariate models, these values were log‐transformed.

Finally, we again clustered trial effect sizes and plotted their reported effect sizes against the re‐estimated effect sizes. Only in this instance, comparator values (pharmacological support, number of BCTs, adjuvant interventions and exposure time) were not based on one of the seven prototypes, but on the average observed comparator values in groups using one of the five prototypical experimental interventions. In other words, all trials were compared with the typical (or average) comparator group used in trials evaluating (1) self‐help; (2) physician advice; (3) nurse/pharmacist counselling; (4) psychologist individual; or (5) psychologist group counselling interventions. If comparators differ systematically between the five prototypical experimental interventions (e.g. between self‐help and group interventions), these meta‐analyses show how conclusions about their (relative) effectiveness change when comparator variability is accounted for. Similar to the step before, estimated ratios were calculated by dividing the predicted cessation rate of the experimental intervention by the predicted cessation rate of the average comparator per trial and then calculating a mean and corresponding 95% CI.

## RESULTS

**FIGURE 2:**
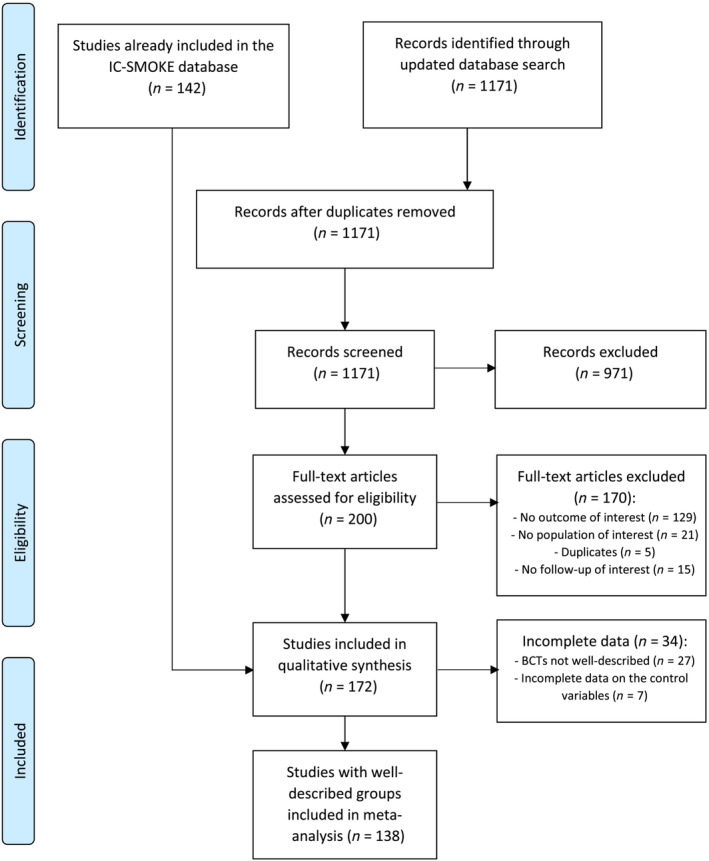
Flowchart of study selection process.

In total, 172 studies met inclusion criteria. Following our author contact protocol and after extracting relevant data from all published and unpublished trial materials, 138 trials had complete data on interventions, covariates and outcomes for inclusion in the meta‐analyses (for study flowchart, see Figure 2). A complete reference list of studies included in this review can be found in Appendix S2.

### Descriptive statistics

The 138 studies (including *N* = 56 729 participants) contained 450 timepoints (range = 22–209 weeks post‐randomisation) and 277 well‐described groups, of which 150 groups were experimental (*n* = 26 984) and 127 were comparator groups (*n* = 29 745). In the experimental groups, high variability was found for the number of total BCTs provided (Mean (M) = 21.47, SD = 11.56, range = 1–53), both for personalised BCTs (M = 7.09, SD = 5.91, range = 0–30) and for non‐personalised BCTs (M = 14.37, SD = 9.19, range = 0–41). The same was observed for the comparator groups (total BCTs M = 15.87, SD = 13.23, range = 0–45; personalised BCTs, M = 2.95, SD = 3.97, range = 0–17; and non‐personalised BCTs, M = 12.91, SD = 10.92, range = 0–42). On average, experimental groups received 241 minutes of exposure time (SD = 321, range = 4–1440), whereas comparator groups had an average exposure time of 127 min (SD = 260.4, range = 1–1440). Sixty‐four per cent of the experimental and 44% of the comparator groups received pharmacological support and 15% of the experimental and 2% of the comparator groups adjuvant interventions, such as physical activity or diet support. Note that experimental and comparator characteristics in trials were associated: when interventions contained more BCTs, pharmacological support, adjuvant interventions or increased exposure time, so did their comparators (all *P*’s < 0.001). In other words, more sophisticated and resource‐intensive experimental interventions were more likely to be compared against more sophisticated and resource‐intensive comparators (and vice versa, simple brief interventions with even simpler and briefer comparators).

### Predicting smoking cessation rates in the comparator and experimental groups: building the model

We ran the pre‐defined models to test the hypotheses for answering the research questions shown in Figure 1 (top row). The decisions after each analysis step are shown in the bottom row of Figure 1. Table SB1 and Supplemental Information SB3 contain all the model output and a detailed explanation of the decisions taken to arrive at the final model. The final model itself is shown in Table 3. We used this final model to estimate the smoking cessation rates in the interpersonal experimental and comparator arms and regressed these on the observed smoking cessation rates (Figure SB4). This model explained approximately half of the variance in smoking cessation rates (pseudo R^2^ = 0.44, *r* = 0.66).

**TABLE 3:** Final model used for re‐estimation of smoking cessation rates.

Predictor	B (SE)
Total BCTs	0.014 (0.004)***
Pharmacological support	0.317 (0.083)***
Exposure time	0.051 (0.022)*
Adjuvant interventions	0.354 (0.097)***
Physical condition	−0.271 (0.169)
Mental health challenge	−0.590 (0.173)***
Attrition	−1.508 (0.287)***
Physical health trigger	0.584 (0.169)***
Age	0.008 (0.007)
Nicotine dependence	−0.067 (0.066)
Log‐transformed length to follow‐up	−0.125 (0.047)**
Cotinine verification	−0.393 (0.106)***
Type of abstinence assessed	−0.237 (0.224)
Studies^a^	116
Groups	221
Timepoints	353

Abbreviations: B, unstandardised β‐coefficient; BCTs, behaviour change techniques; SE, standard error.

^a^ Model size reduced since exposure time is included in the model, but exposure time is unknown for written (i.e. self‐help) groups and is coded with NA (not available) in these groups. Self‐help groups are, therefore, dropped from the final model.

* *P* < 0.05;

** *P* < 0.01;

*** *P* < 0.001.

### Re‐estimation of cessation rates and comparison of prototypical interventions

The next step was to re‐estimate effect sizes of all trials, as if all interventions had been compared against the same prototypical comparators. Although the final model (Table 3) is based on trials evaluating in‐person delivered interventions so that exposure time can be included, the active content estimates fall well within the 95% CI of the model for written interventions (Tables SB2 and SB3). We, therefore, used the final in‐person delivery model to also re‐estimate the effects for written interventions. Below, we compare trials’ reported effect size with seven re‐estimated effect sizes based on the comparator prototypes (Table 2).

**FIGURE 3:**
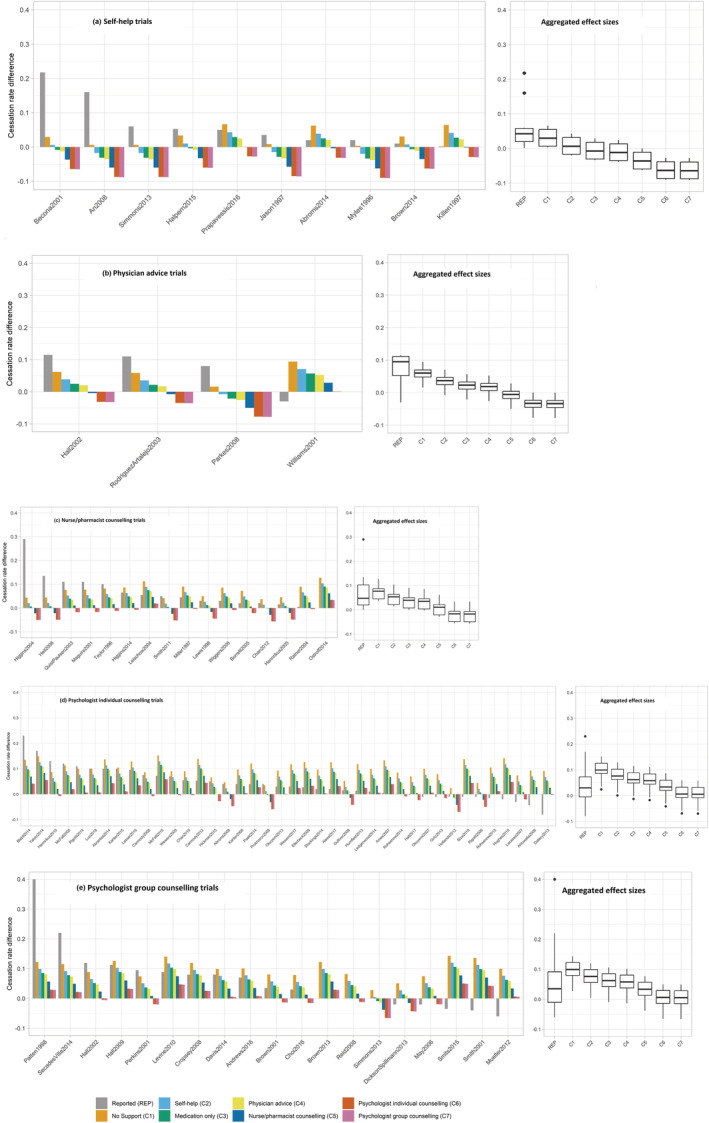
There are five panels for different intervention prototypes. Within each panel, the barplots within each panel show for each trial the cessation rate difference (effect size) actually reported in trial papers shown in the grey bar, followed by seven and predicted cessation rate differences of each individual trial and for each combination of five prototypical experimental and reflecting the estimated effect of the individual experimental interventions against each of the seven prototypical comparator groups (C1–C7). The eight bars within each cluster of trials represent the actually reported (grey bar) and predicted (other colours) cessation rate differences for experimental versus comparator group within each specific trial. Within a panel, trials are ordered according to actually reported cessation rate differences reported in the trial paper (highest to lowest). The predicted cessation rate differences represent the differences in the predicted cessation rates of experimental interventions relative to all seven standardised prototypical comparator groups. The boxplots next to each panel show the aggregated effect sizes for each of the coloured bars. The first is for the actually difference scores of reported cessation rate differences, followed of each prototypical experimental intervention (left box within each boxplot) and aggregated difference scores of by the seven aggregated predicted cessation rate differences for the re‐estimated effect sizes between prototypical experimental interventions and prototypical comparator groups (other seven boxes within each boxplot). The black line within each box represents the median. The boxplots represent the predicted average difference in smoking cessation rates when comparing the cessation rates of the five experimental groups with the seven standardised comparator groups. For the prediction of cessation rates, covariates that were not considered active content were held constant at a fixed value. Adjuvant interventions, physical condition, mental health challenges and physical health trigger were fixed to zero, because the majority of groups in the analyses scored had zero on these variables. Attrition was fixed to zero. Age was fixed to 43.46, because this was the mean age of all groups included in the analyses. Nicotine dependence was set to 4.89, because this was the mean score on the Fagerström Test for Nicotine Dependence of all groups included in the analyses. Length to follow‐up was fixed to 26 weeks.

Figure 3 shows five panels including bar charts of the reported and re‐estimated effect sizes for all trials, with trials grouped for the five intervention prototypes. The trials are organised from left to right by the size of their reported effect size. The left bar within each trial reflects the reported relative smoking cessation rate in the trial, followed by the seven re‐estimated effect sizes ordered from minimal (no support) to the most intensive (group counselling) prototypical comparator. On the right side of each panel, the aggregated effect size for that group of interventions is shown in boxplots: the reported effect sizes and re‐estimated for the seven prototypical comparators.

Figure 3 illustrates three key points. First, at the level of individual trials, if you compare the trials by their reported effect size (take the individual nurse counselling trials in Figure 3C) and then examine their re‐estimated effect sizes after standardising the comparator (take the ‘no support’ comparator), conclusions about which trial tests the most promising intervention can change substantially. This shows that effect sizes of trials evaluating the same type of intervention cannot be directly compared without detailed knowledge of their comparators. Note that there are some reported effect sizes (trials on the left) that are much larger than the re‐estimated effect size with a ‘no support’ comparator. This may be because of an exceptional synergy of intervention components and/or sample characteristics not captured by our model, reporting bias or chance (e.g. sample sizes of these trials tend to be small). High‐quality replications of those intervention trials seem warranted.

Second, as we move from left to right through the seven re‐estimated effect sizes within each trial, effect sizes become smaller and often even negative—in particular for scenarios in which self‐help or brief interventions are compared with elaborate comparators more commonly used in individual or group counselling trials (mainly in Figure 3A–C). This is also evident in the aggregated effect sizes for each prototypical intervention (boxplots within each panel), which includes first the reported and then the seven re‐estimated effect sizes for each prototypical intervention. For self‐help interventions, for example, the aggregated reported effect size is 0.06 (95% CI = 0.02–0.11), but the re‐estimated effect size against the comparator using group counselling is negative (−0.06 [95% CI = −0.08 to −0.05]).

Third, the reported effects of the more elaborate and resource‐intensive psychologist counselling interventions are considerably smaller than their re‐estimated effects against the minimal comparator conditions. Hence, because of the design choice to evaluate more potent experimental interventions against more potent comparators, the reported effect sizes increasingly deviate from interventions’ ‘true effectiveness’ as smoking cessation interventions become more resource intensive.

The points described above also complicate comparisons on the (cost) effectiveness of different intervention prototypes, for example, how many additional people quit smoking following more costly psychologist group counselling interventions compared with cheaper self‐help interventions. If you look at the aggregated reported effect sizes for each prototypical intervention, average smoking cessation rates between clusters of trials are relatively comparable.

However, when we standardise comparators to, for example, the ‘no support’ prototype, we estimate that smoking cessation rates are 1.33 (95% CI = 1.16–1.49), 1.61 (95% CI = 1.31–1.90) and 1.76 (95% CI = 1.62–1.90) times higher for self‐help, brief physician advice and nurse/pharmacist counselling interventions, respectively, and 2.04 (95% CI = 1.95–2.15) and 2.06 (95% CI = 1.92–2.20) times higher for psychologist individual and group counselling interventions. An overview of all ratio estimates can be found in Table 4. Note that when the experimental and comparator interventions have the same label based on their mode of delivery (e.g. self‐help, psychologist individual counselling), on average, the experimental interventions tend to be more intensive and contain more BCTs. Hence, re‐estimated effect sizes for experimental interventions are higher than those for comparators with the same label.

**TABLE 4:** Ratio estimates for all combinations of the five experimental versus seven prototypical comparator interventions based on predicted effect sizes and the average active content values of comparator and experimental interventions.

	Prototypical comparator						
	No support	Self‐help	Medication only	Physician advice	Nurse counselling	Individual counselling	Group counselling
Self‐help	1.33 [1.16; 1.49]	1.07 [0.83; 0.95]	0.95 [0.83; 1.07]	0.92 [0.81; 1.04]	0.78 [0.69; 0.88]	0.67 [0.59; 0.75]	0.67 [0.58; 0.75]
Physician advice	1.61 [1.31; 1.90]	1.29 [1.05; 1.53]	1.15 [0.94; 1.37]	1.12 [0.91; 1.32]	0.95 [0.77; 1.12]	0.81 [0.66; 0.95]	0.81 [0.66; 0.96]
Nurse counselling	1.76 [1.62; 1.90]	1.42 [1.30; 1.53]	1.27 [1.17; 1.37]	1.23 [1.13; 1.32]	1.04 [1.22; 0.96]	0.89 [0.82; 0.96]	0.89 [0.82; 0.96]
Individual counselling	2.04 [1.95; 2.15]	1.64 [1.56; 1.72]	1.47 [1.40; 1.54]	1.42 [1.35; 1.49]	1.21 [1.15; 1.27]	1.03 [0.98; 1.08]	1.02 [0.98; 1.08]
Group counselling	2.06 [1.92; 2.20]	1.65 [1.54; 1.77]	1.48 [1.38; 1.58]	1.43 [1.33; 1.53]	1.22 [1.13; 1.30]	1.03 [0.98; 1.08]	1.02 [0.98; 1.08]

*Note*: Estimates <1 indicate that the comparator intervention is more effective than the experimental intervention.

**FIGURE 4:**
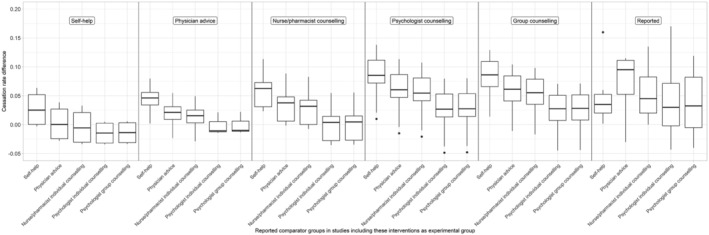
Aggregated predicted cessation rate differences for the five prototypes of experimental interventions using the average reported active content that comparator groups within each of the trials including these prototypical experimental interventions actually used. For example, for all trials including self‐help as an experimental group, the average reported active content of the comparator groups actually used in these studies was calculated (total number of behaviour change techniques, medication and exposure time). These average values were then used to predict cessation rates of the comparator groups for all trials using self‐help interventions as experimental group. Afterwards, the difference in the predicted cessation rates of the experimental and comparator groups was calculated. The same was done for the other prototypes of experimental interventions. The plot illustrates the predicted cessation rate difference between experimental and comparator groups without standardising comparator groups, but rather using the characteristics of comparator groups that were actually used within each cluster of prototypical experimental interventions. Note, the black line within each box represents the median.

In reality, if you combine all trials that evaluate a prototypical intervention—like self‐help or psychologist individual counselling—they are using various comparator prototypes. We, therefore, calculated the average ‘average comparator’ active content used in all groups that used the five experimental intervention prototypes, re‐estimated effect sizes for all trials against these average values, aggregated those and compared them with the aggregated reported effect sizes for each intervention prototype (see Figure 4). Instead of assuming that prototypical experimental interventions are always compared to only one prototypical comparator, as we did in the previous step, this gives a clearer picture of how comparators actually used in trials distort findings on relative effectiveness of smoking cessation interventions.

The patterns are very similar to those reported in the previous paragraph: the reported effect sizes give a distorted image of the relative effectiveness of intervention prototypes because of variability in the comparators used. For example, when using the average comparator values used in trials evaluating self‐help interventions, psychologists group counselling and individual counselling interventions appear 1.79 (95% CI = 1.67–1.92) and 1.78 (95% CI = 1.70–1.87) times more effective. This is very different from the conclusions one would draw when examining the reported effect sizes (right panel, Figure 4). An overview of these ratio estimates can be found in Table 5.

**TABLE 5:** Ratio estimates of average comparators actually used in trials of five prototypical experimental interventions versus effectiveness of experimental intervention prototypes.

	Average comparator actually used in trials of prototypical experimental interventions				
	Self‐help	Physician advice	Nurse counselling	Individual counselling	Group counselling
Self‐help	1.24 [1.09; 1.39]	1.01 [0.89; 1.14]	0.97 [0.85; 1.09]	0.81 [0.71; 0.91]	0.81 [0.71; 0.91]
Physician advice	1.39 [1.14; 1.66]	1.14 [0.93; 1.35]	1.09 [0.89; 1.29]	0.91 [0.74; 1.08]	0.91 [0.75; 1.08]
Nurse counselling	1.53 [1.41; 1.66]	1.25 [1.15; 1.35]	1.20 [1.10; 1.29]	1.00 [0.92; 1.08]	1.00 [0.92; 1.08]
Individual counselling	1.78 [1.70; 1.87]	1.45 [1.38; 1.52]	1.39 [1.32; 1.46]	1.16 [1.10; 1.21]	1.17 [1.11; 1.22]
Group counselling	1.79 [1.67; 1.92]	1.46 [1.36; 1.56]	1.40 [1.30; 1.50]	1.17 [1.09; 1.25]	1.17 [1.09; 1.26]

*Note*: Estimates <1 indicate that the comparator intervention is more effective than the experimental intervention.

## DISCUSSION

The results demonstrate that variability and underreporting of comparators obscure the interpretation, comparison and generalisability of behavioural smoking cessation trials. When we re‐estimate the effectiveness of individual trials against standardised comparators, or the (relative) effectiveness of prototypical interventions, conclusions about which interventions appear most effective change considerably. First, when accounting for variability in comparators, trials that based on their reported results appear to be the most effective in their category (e.g. individual nurse counselling interventions) can be among the least effective when variability in comparators is accounted for. Second, experimental groups receiving simple, brief interventions appear worse off than comparator groups in trials evaluating more elaborate, intensive interventions; and vice versa, more complex interventions that appear of limited use based on their reported effects appear highly effective against the comparators used in trials evaluating simple interventions. Finally, the relative effectiveness of different prototypical interventions based on the aggregated reported effect sizes changes quite drastically when the comparator is standardised. Hence, incomplete reporting of experimental and comparator interventions, and not accounting for variability in comparators when interpreting and comparing effect sizes of smoking cessation intervention trials, is likely to lead to inaccurate conclusions.

This study was designed to be a large‐scale, more rigorously conducted confirmatory study of a prior small‐scale meta‐analysis we conducted in the area of HIV treatment behaviours [6]. That very similar results were obtained in the current study on smoking cessation (a prevention behaviour) suggests that this issue may apply to behavioural intervention trials more generally (at least those with active comparators). Hence, it appears that the value of behavioural intervention trials and their evidence syntheses could improve considerably if comparators were to be much more comprehensively reported and that differences in comparators were to be taken into account in systematic reviews and meta‐analyses of these trials. This could contribute to a more robust science and better‐quality input for healthcare policymaking and practice. We cannot sufficiently stress the importance of comprehensive reports of comparator interventions in behavioural trials (but also other trials of more complex interventions) at the same level of detail as the experimental group [7, 21]. This would make it possible for readers, systematic reviewers and decision makers (policymakers, practitioners) to incorporate that in their decision making and modelling.

### Implication for smoking cessation

The current study focused on behavioural smoking cessation trials, selected studies using objective outcomes for smoking cessation, collected all published data and a large amount of unpublished data from trial authors, used pre‐registered and hypothesis‐driven analyses plans for the main variables as well as the control variables and accounted for comparator variability when comparing trials and intervention categories. For smoking cessation researchers, practitioners and policymakers, it offers several contributions to the current literature. First, the re‐estimated trial effect sizes in Figure 3 can help identify the most promising smoking cessation interventions to adopt into policy or practice, in terms of their estimated effectiveness under different comparator (including ‘usual care’) conditions. Figure 3 also shows that interventions that on first sight appear highly effective in helping people stop smoking (usually the one or two trials on the left‐hand side in Figure 4 panels) appear as outliers based on their reported smoking cessation rates and are rarely among the more effective interventions under standardised comparator conditions (and standardised covariates). It, therefore, seems important that these potentially very promising interventions are replicated in another trial before being considered for adoption in guidelines and implemented in practice. Third, Figure 3 also shows that when more resource‐intensive smoking cessation interventions (i.e. nurse and in particular psychologists individual and group counselling comparators) are replaced with ‘light’ interventions (such as brief advice or self‐help), we can expect to see a substantial decrease in the effectiveness of the smoking cessation services provided.

Figure 4 shows that when you aggregate and compare interventions based on their reported effectiveness, there is actually not a good argument for investing in more resource‐intensive smoking cessation interventions. All types of interventions appear equally effective. However, this turns out to be because more resource‐intensive and effective interventions are compared with more resource‐intensive and effective comparators. When comparators are standardised, however, it becomes evident that psychologist and group counselling interventions are on average three times as effective as self‐help interventions, almost twice as effective as brief physician advice and 1.5 times as effective as the medium‐intensive non‐specialist nurse/pharmacist counselling interventions. Hence, policymakers and practitioners are advised to—when they decide on which smoking cessation services to offer—account for differences in comparators between intervention categories (e.g. by using the results of the current meta‐analysis or network meta‐analyses that successfully account for comparator variability).

The Cochrane Tobacco Addiction Group regularly conducts state‐of‐the‐art systematic reviews and meta‐analyses of smoking cessation interventions, which—as discussed in the introduction—may account for comparator variability to some extent. If we compare the results of the current study with those reviews, there are a number of similarities and differences. Most notable, the findings from the pairwise meta‐analyses are consistent with our results in the sense that as the comparator becomes more intensive (e.g. from no intervention to self‐help to brief and then intensive longitudinal counselling), the effectiveness of the type of intervention evaluated decreases. Comparing results to the network meta‐analysis [19], the direction of effects of intervention components was usually similar although there were differences in significance. This could be because of differences in sample size, statistical technique and differences in the completeness of data describing the experimental and comparator interventions [5]. What the current study, using an approach that is similar to a component network meta‐analysis, adds to this is that it identifies intervention components in experimental and comparator arms predicting smoking cessation using comprehensive data on the content of interventions and that it uses those data to estimate the true effectiveness of individual and prototypical interventions against a range of prototypical comparators. That is particularly relevant given that comparators vary widely between individual trials and between intervention prototypes.

### Strengths and limitations

The strengths of the current systematic review and meta‐analysis are that we used a very comprehensive dataset of behavioural smoking cessation trials, collected a large amount of missing intervention data from trial authors, focused on trials using objective outcomes, systematically identified control and predictor variables based on literature and expert input, pre‐published detailed analyses plans and conducted a range of model checks to ensure reliability. Another strength was that this study was designed as a high‐quality, well‐powered replication study of an earlier, much smaller meta‐analysis without many of these methodological strengths [6]. Potential limitations are that the active content predictors were—necessarily—a simplification as different BCTs, different smoking cessation medications and different adjuvant interventions were grouped into single variables. Another limitation is that the literature search is almost 4 years old, meaning that more recent smoking cessation trials could not be re‐estimated in our analyses. However, for most intervention groupings, there were a substantial number of included trials, and the absence of the most recent studies is unlikely to have affected the conclusions regarding the main methodological questions examined in this paper. Finally, in the absence of adequate reporting of experimental and comparator interventions, as indicated by CONSORT (for social and pychological interventions; SPI) and TIDieR statements, collecting comprehensive data on experimental and comparator interventions is a time‐consuming task. However, in the absence of other indicators to capture this variability, it appears a necessary step to arrive at more reliable estimates of the (relative) effectiveness of smoking cessation interventions.

### CONCLUSION

Underreporting of experimental and in particular comparator interventions in behavioural smoking cessation trials, and not fully accounting for comparator variability in systematic reviews and meta‐analyses, may lead to invalid conclusions about the relative effectiveness of different types of smoking cessation interventions and what individual interventions are most promising. After accounting for comparator variability, this meta‐analysis showed that psychologist and group counselling interventions are on average three times as effective as self‐help interventions, almost twice as effective as brief physician advice and 1.5 times as effective as the medium‐intensive non‐specialist nurse/pharmacist counselling interventions. Not adjusting for comparator variability leads to overestimation of effects of relatively simple and cheap interventions and underestimation of effects of more complex and labour‐intensive interventions.

## AUTHOR CONTRIBUTIONS

**Jannis Kraiss:** Conceptualization; formal analysis; methodology; visualization; writing—original draft; writing—review and editing. **Wolfgang Viechtbauer:** Conceptualization; formal analysis; methodology; software; writing—review and editing. **Nicola Black:** Conceptualization; data curation; formal analysis; writing—review and editing. **Marie Johnston:** Conceptualization; supervision; writing—review and editing. **Jamie Hartmann‐Boyce:** Conceptualization; supervision; writing—review and editing. **Maarten Eisma:** Conceptualization; supervision; writing—review and editing. **Neza Javornik:** Conceptualization; data curation. **Alessio Bricca:** Conceptualization; data curation; writing—review and editing. **Susan Michie:** Conceptualization; supervision; writing—review and editing. **Robert West:** Conceptualization; supervision; writing—review and editing. **Marijn de Bruin:** Conceptualization; formal analysis; funding acquisition; project administration; supervision; writing—original draft; writing—review and editing.

## DECLARATION OF INTERESTS

None.

## REGISTRATION

PROSPERO (CRD42015025251) and the Open Science Framework (https://osf.io/23hfv/).

### OPEN RESEARCH BADGES

Open Data Badge (https://osf.io/uvemc) and Preregistered Badge (https://osf.io/khm8u).

## Supporting information


**Appendix S1** Additional methodological information.
**Appendix S2** Additional results information.

## Data Availability

Data is available on the OSF website of this project (https://osf.io/23hfv/).
